# Development of Data Registration and Fusion Methods for Measurement of Ultra-Precision Freeform Surfaces

**DOI:** 10.3390/s17051110

**Published:** 2017-05-12

**Authors:** Ling Bao Kong, Ming Jun Ren, Min Xu

**Affiliations:** 1 Shanghai Engineering Research Center of Ultra-Precision Optical Manufacturing, Fudan University, Shanghai 200433, China; LKong@fudan.edu.cn (L.B.K.); minx@fudan.edu.cn (M.X.); 2 Institute of Robotics, School of Mechanical Engineering, Shanghai Jiao Tong University, Shanghai 200240, China

**Keywords:** data fusion, data registration, intrinsic surface features, ultra-precision freeform surfaces, precision metrology

## Abstract

The measurement of ultra-precision freeform surfaces commonly requires several datasets from different sensors to realize holistic measurements with high efficiency. The effectiveness of the technology heavily depends on the quality of the data registration and fusion in the measurement process. This paper presents methods and algorithms to address these issues. An intrinsic feature pattern is proposed to represent the geometry of the measured datasets so that the registration of the datasets in 3D space is casted as a feature pattern registration problem in a 2D plane. The accuracy of the overlapping area is further improved by developing a Gaussian process based data fusion method with full consideration of the associated uncertainties in the measured datasets. Experimental studies are undertaken to examine the effectiveness of the proposed method. The study should contribute to the high precision and efficient measurement of ultra-precision freeform surfaces on multi-sensor systems.

## 1. Introduction

Ultra-precision freeform surfaces with sub-micrometer-form accuracy and surface finish in the nanometric range are now widely adopted in opto-mechatronic applications, due to their superior mechanical and optical properties in improving the performance of the products in both functionality and size reduction [1]. The trend of miniaturization has further driven the integration of multi-scale representations of features in single surfaces which exhibit specific functionalities at different scales [2,3]. However, the geometric complexity also brings about many challenges for the measurement of these surfaces, especially large-sized and multi-scaled freeform surfaces.

In the past decades, many precision measurement instruments have been developed with respect to specific and varying metrological needs. Many existing precision measurement instruments only possess limited measuring range at one single measurement, and are difficult to perform the high precision measurement of large size with both high resolution and efficiency. This is particularly true for some freeform surfaces with high slopes or large sizes [4,5]. Multi-sensor instruments have been considered as a promising solution for measuring these kinds of surfaces [6]. Several different sensors are integrated into a single instrument to perform cooperative measurements, so as to enhance measurement range and fidelity, while minimizing measurement cost and time. For example, Werth VideoCheck UA 400 [7] integrates an imaging sensor, tactile scanning sensor, and white light sensor into a single system which is capable of measuring complex 3D geometries with sub-micrometric accuracy. WITec GmbH [8] integrates confocal Raman microscopy, atomic force microscopy, and scanning near-field optical microscopy, and can perform relatively fast measurements of large-area samples. However, these instruments simply integrate several sensors into a common system, and lack the much-required multi-sensor data fusion functionality and characterization that would achieve improved measurement results.

Multi-sensor datasets may come from different spaces with different scales, resolutions, and associated uncertainties. The effectiveness of the multi-sensor metrology heavily depends on the quality of the data registration and fusion, which are further steps for sensor integration, and are responsible for combining the measured datasets from different sensors into a common representation, in order that the measurement can benefit from the technical merits of all the involved sensors. Data registration and fusion has been an emerging technology since the 1990s, and it has previously been used for target tracking, automated identification of targets, and limited automated reasoning applications [9]. The technology is then adopted in reverse engineering and precision metrology [10]. Generally, for surface measurement, the process includes pre-processing, data registration, data fusion and post processing, among which data registration and fusion are the most critical steps [6]. In registration, all the datasets are transformed to a common coordinate frame based on rigid motion. Due to geometry complexity and variety, as well as a lack of common features for freeform surfaces, it is still very difficult to register one freeform surface to another with high precision in the presence of noise. An open literature review shows that data registration can be realized by feature-based or surface description-based approaches, and the registration process generally includes coarse and fine registration [11]. Some research work has been found for data stitching of aspherical surfaces [12,13]. The iterative closest point (ICP) method is often used for correspondence searching [14]. However, this is susceptible to data noise, and outliers are involved in the measured data, and accumulative errors would be produced when a large amount of datasets are involved [15]. There is still little research into data registration and fusion of ultra-precision freeform surfaces with sub-micrometer form accuracy.

Fusion is responsible for processing the redundant data in the overlapping area of the datasets. Considering that the fused datasets may have different resolutions with different associated uncertainties, proper fusion process should be carried out to fuse the datasets which may have different resolutions with different associated uncertainties. Wang et al. [16] reviewed current data fusion methods in surface metrology, and summarized the data fusion methods into four categories, including repeated measurements, stitching, range image fusion, and 3D data fusion. Ramasamy et al. [17] presented several data fusion strategies and weighting methods in the fusion of multi-scaled range images. Although the validity of the method has been confirmed for the measurement of micro-structured surfaces, the uncertainty propagation is not clearly demonstrated. Forbes et al. [18] presented a weighted least square-based data fusion method that relies on linear approximation of the geometry of the datasets. The method may be problematic when the datasets have sharp geometrical changes, for instance, a smooth surface embedded with micro-structures.

This paper presents a study of data registration and fusion for measuring ultra-precision freeform surfaces on multi-sensor instruments. The method performs the data registration by representing the geometry of each dataset based on intrinsic surface features which are invariant to coordinate transformation, and free from the implicit parameterization of the surface. Data fusion is then performed at the overlapping area based on a Gaussian process model, to further reduce the measurement uncertainty. Experimental studies are presented to demonstrate the validity of the proposed method.

## 2. Data Registration and Fusion Methods

Intrinsic surface features (ISFs) refer to those surface features whose values are invariant under the transformation (rotation/translation) of the embedded coordinate frame, and also free from the implicit parameterization of the surface. Surface registration can be performed without the need to consider the misalignment of the coordinate frames and the parameterization of the surfaces when the two surfaces are represented by ISFs. Two of the important ISFs are Gaussian and mean curvature. They uniquely determine the surface shape according to the Gaussian Curvature Uniqueness Theorem and the Mean Curvature Uniqueness Theorem [19,20].

**Figure 1:**
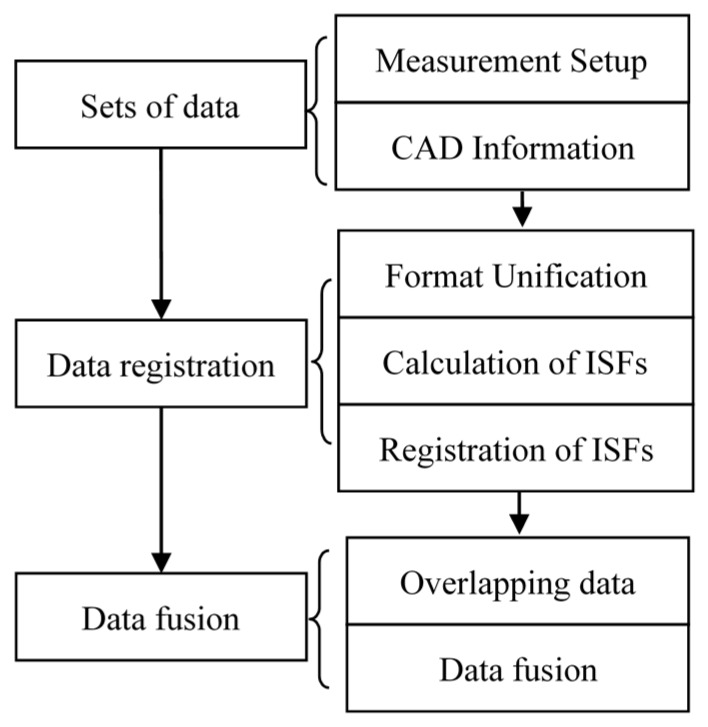
Flow chart of data registration and fusion.

**Figure 2:**
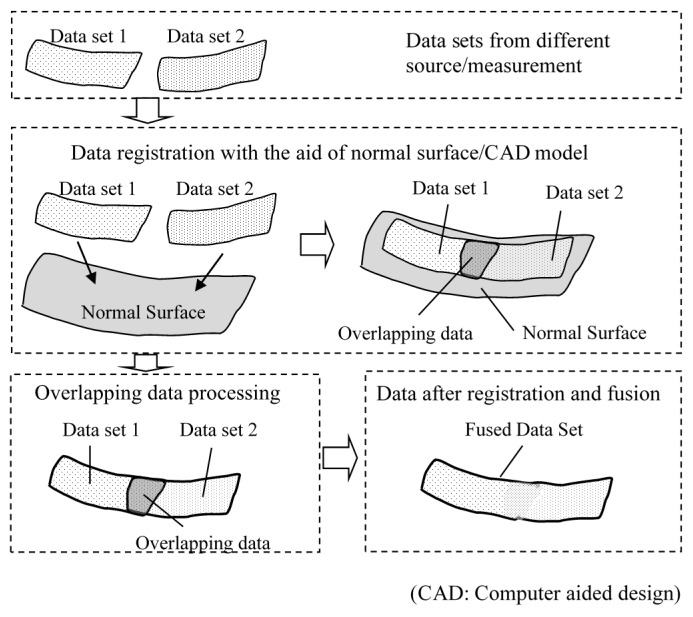
Graphical illustration of data registration and fusion.

Figure 1 shows a flow chart of the proposed data registration and fusion method. Different sets of data were obtained with measurement setup information, and the surface normal/computer aided design (CAD) model was provided. The data format was unified, and hence the ISFs of the data sets were calculated, followed by registration to find the correspondence. The overlapping area among the datasets was then identified, and data fusion was carried out. Figure 2 illustrates the data registration process with the aid of the nominal surface/CAD model. The normal surface or CAD model information was provided, and different sets of data were registered to the normal surface or CAD surface firstly, and hence they were stitched to each other. Figure 2 illustrates data stitching process with the aid of normal surface/CAD model. If there was no CAD information, different sets of data were directly registered to each other by maximizing the similarity of their overlapping areas. Some important algorithms are explained in detail in the coming sections.

### 2.1. Data Reformat by Re-Sampling

**Figure 3:**
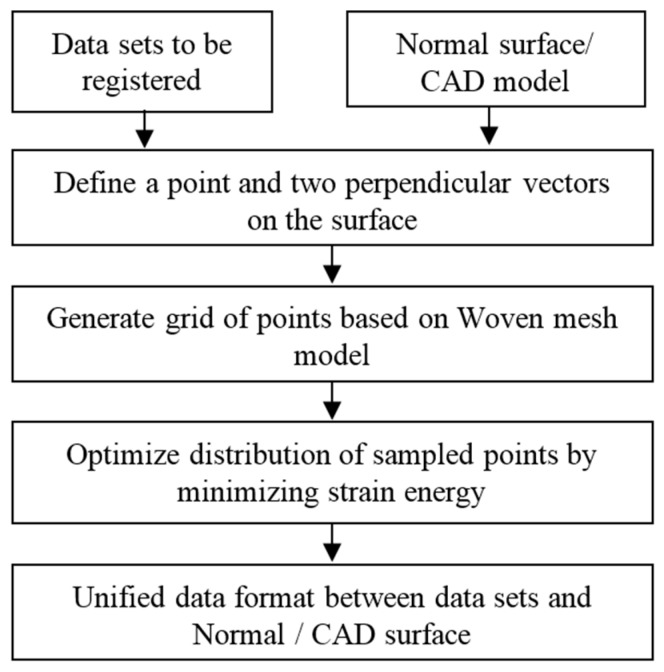
Flow chart for data format unification.

Before registration of the datasets, the format of different datasets was unified by a re-sampling strategy. This was realized by the process as shown in Figure 3. A grid of points were uniformly sampled on a dataset and the values of the ISFs of these points were arranged on a two dimensional (2D) plane to form an intrinsic feature pattern. The pattern was not only invariant to the coordinate transformation but was also free from the implicit parameterization of the surface. The layout of a 2D texture onto a general freeform surface inevitably creates distortion in all but developable surfaces, i.e., surfaces with zero Gaussian curvature, such as a cylinder [19]. Hence, the problem was concerned fitting a 2D pattern into a freeform surface such that the texture distortion was minimized. In the present study, a woven mesh model [21] was employed to address this problem. Woven mesh model is a kind of woven fabric model, which consists of several types of springs in different directions. These springs have their own initial length at which the spring has zero energy. When the mesh model is fitted into a freeform surface, the texture may be distorted and the directions and lengths of the springs are not preserved as compared with the original 2D pattern. This leads to the strain energy. The distortion can then be minimized by minimizing the strain energy in the mesh model, through optimizing the distribution of the points. More details of the woven mesh model can be found in [21]. After that, the format of datasets and normal surface were unified for later registration based on ISF.

### 2.2. Calculation of Intrinsic Surface Features

In the present research, Gaussian curvature or mean curvature was chosen as the ISF. Direct calculation of the ISF, such as Gaussian curvature, from the measured datasets is sensitive to the measurement noise. Hence, in the present study, the B-spline surface was used to fit the discrete points so that the measurement noise could be rejected during the surface fitting process and the ISF could be safely calculated [22]. Suppose that S(u,v) is the fitted B-spline surface, where (u,v) are the parameters of the surface; the first fundamental form of S(u,v) can be expressed as

(1) $$I=Edu^{2}+2Fdudv+Gdv^{2}$$

where

(2) $$\left\{ \begin{array}{l} E=S_{u}^{2}={\left(\frac{\partial S(u,v)}{\partial u}\right)}^{2}\\ F=S_{u}S_{v}=\frac{\partial S(u,v)}{\partial u}\frac{\partial S(u,v)}{\partial v}\\ G=S_{v}^{2}={\left(\frac{\partial S(u,v)}{\partial v}\right)}^{2} \end{array}\right.$$

The second fundamental form of S(u,v) is given by

(3) $$II=Ldu^{2}+2Mdudv+Ndv^{2}$$

where,

(4) $$\left\{ \begin{array}{l} L=\frac{\partial^{2}S(u,v)}{\partial u^{2}}\cdot \frac{S_{u}\times S_{v}}{\left\|S_{u}\times S_{v}\right\|}\\ M=\frac{\partial^{2}S(u,v)}{\partial u\partial v}\cdot \frac{S_{u}\times S_{v}}{\left\|S_{u}\times S_{v}\right\|}\\ N=\frac{\partial^{2}S(u,v)}{\partial v^{2}}\cdot \frac{S_{u}\times S_{v}}{\left\|S_{u}\times S_{v}\right\|} \end{array}\right.$$

Then, Gaussian curvature *K* and mean curvature *H* of the surface S(u,v) are determined by the coefficients of the first and second fundamental forms as follows, respectively.

(5) $$K=\det\left({\left[\begin{array}{cc} E & F\\ F & G \end{array}\right]}^{-1}\right)\det\left(\left[\begin{array}{cc} L & M\\ M & N \end{array}\right]\right)$$

(6) $$H=\frac{1}{2}tr\left({\left[\begin{array}{cc} E & F\\ F & G \end{array}\right]}^{-1}\right)\det\left(\left[\begin{array}{cc} L & M\\ M & N \end{array}\right]\right)$$

where operator det( ) is the determinant of a matrix; operator tr( ) is the trace of a matrix.

### 2.3. Registration Process Based on ISF

**Figure 4:**
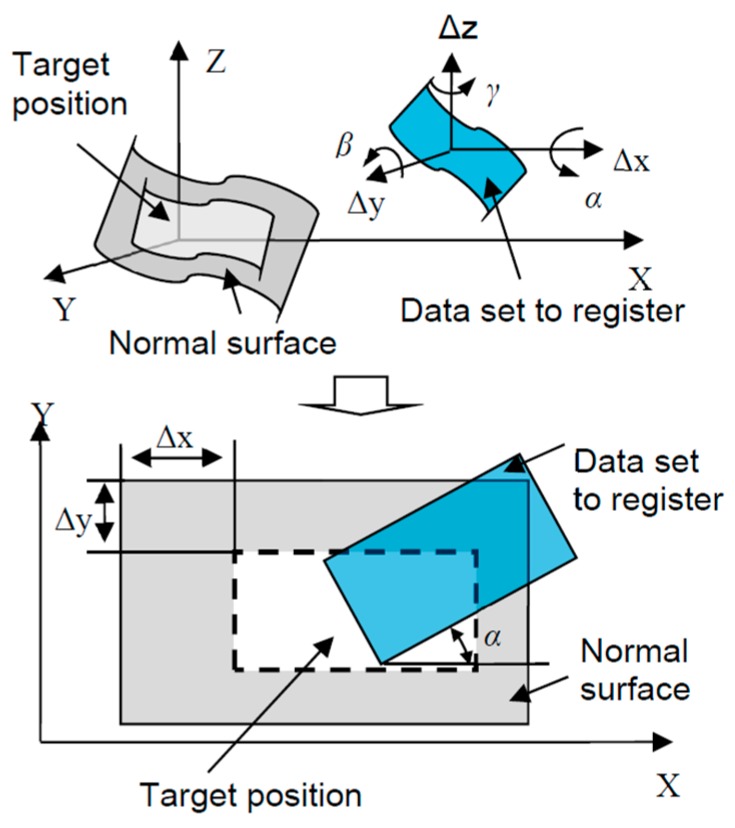
Illustration of data registration based on ISFs.

The surface registration problem was then converted to ISF registration. That is, the corresponding searching in 3D Cartesian coordinate frame by solving six parameters (three translations plus three rotations) was now performed in 2D space by finding three parameters (two translations plus one rotation), as shown in Figure 4. Registration problems involving translation and rotation were recovered by applying a Fourier-Mellin transform and the phase correlation method [23].

Suppose $f_{2}\left(x,y\right)$ is translated and rotated from $f_{1}\left(x,y\right)$, then

(7) $$f_{2}(x,y)=f_{1}(x\cos(\alpha )-y\sin(\alpha )-\Delta x,\;x\sin(\alpha )+y\cos(\alpha )-\Delta y)$$

where Δx and Δy are the translation offsets and α is the rotation angle. According to the Fourier translation property and the Fourier rotation property, the Fourier transformation of $f_{1}$ and $f_{2}$ are related by

(8) $$F_{2}(\xi ,\eta )=\exp(-j2\pi (\xi \Delta x+\eta \Delta y))\cdot F_{1}(\xi \cos(\alpha )-\eta \sin(\alpha ),\xi \sin(\alpha )+\mu \cos(\alpha ))$$

where $F_{1}$ and $F_{2}$ are Fourier transform of $f_{1}$ and $f_{2}$, respectively. Therefore, Equation (9) preserves

(9) $$M_{2}(\xi ,\eta )=M_{1}(\xi \cos(\alpha )+\eta \sin(\alpha ),-\xi \sin(\alpha )+\eta \cos(\alpha ))$$

where, $M_{1}\;$and $M_{2}$ are magnitudes of $F_{1}$ and $F_{2}$, respectively.

From Equation (9), the translation is recovered, and the rotation causes the spectral magnitude to be rotated at the same angle, which can be determined in polar coordinates [24].

(10) $$PM_{2}(\rho ,\theta )=PM_{1}(\rho ,\theta -\alpha )$$

where $PM_{1}$ and $PM_{2}$ are the spectral magnitudes of $f_{1}$ and $f_{2}$ in polar coordinates, respectively. The rotating angle can be determined by translation offset in the polar coordinate system by using the phase correlation as follows:

(11) $$Corr(u,v)=\frac{FPM_{1}(u,v)}{\left|FPM_{1}(u,v)\right|}\cdot \frac{FPM_{2}(u,v)}{\left|FPM_{2}(u,v)\right|}=\exp\left(-2\pi (u+v\alpha )\right)$$

where$\;FPM_{1}$ and $FPM_{2}$ are the Fourier transform of $PM_{1}$ and $PM_{2}$. The Inverse Fourier Transform of Equation (11) is a Dirac δ-function yielding a sharp maximum at (0,α). Hence, the $f_{2}$ is rotated by α, and the rotated $f_{2}$ is phase correlated with $f_{1}$ again to determine the translational offsets Δx and Δy.

**Figure 5:**
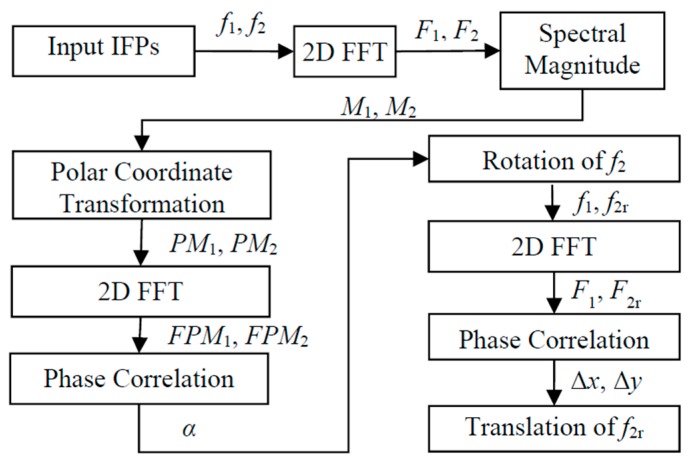
Flowchart of the IFP registration process.

Figure 5 summarizes the algorithms to find the translation and rotation offsets between the two IFPs. It started with inputting two IFPs, i.e., $\left(f_{1},f_{2}\right)$, and the Fourier transform of the two patterns $\left(F_{1},F_{2}\right)$ was computed by 2D fast Fourier transform (2D FFT). The spectral magnitudes $\left(M_{1},M_{2}\right)$ of the two IFPs were then transformed to the polar coordinate system, and the rotation angle was determined by the phase correlation method. The determined rotation angle α was then used to recover the rotation of the $f_{2}$, and the rotated $f_{2}$, denoted as $f_{2r}$, was then used to perform phase correlation again with $f_{1}$, to recover the translation.

The established correspondence by ISF registration was then used to evaluate the coordinate transformation matrix T by minimizing the sum of the squared distance of each correspondence pairs.

(12) $$F=\sum_{k=0}^{n}{\left|X1_{k}-T\;X2_{k}\right|}^{2}$$

(13) $$T\left(r_{x},\;r_{y},\;r_{z},\;t_{x},\;t_{y},\;t_{z}\right)=\left[\begin{array}{cccc} c(r_{z})c(r_{y}) & s(r_{z})c(r_{y})+c(r_{z})s(r_{y})s(r_{x}) & s(r_{z})s(r_{x})-c(r_{z})s(r_{y})c(r_{x}) & t_{x}\\ -s(r_{z})c(r_{y}) & c(r_{z})c(r_{x})-s(r_{z})s(r_{y})s(r_{x}) & c(r_{z})s(r_{x})+s(r_{z})s(r_{y})c(r_{x}) & t_{y}\\ s(r_{y}) & -c(r_{y})s(r_{x}) & c(r_{y})c(r_{x}) & t_{z}\\ 0 & 0 & 0 & 1 \end{array}\right]$$

where $\left(X1_{k},X2_{k}\right)$ are the correspondence pairs, n is the number of the established correspondence pairs, $t_{x}$, $t_{y}$, $t_{z}$ are the translation components, and $r_{x}$, $r_{y}$, $r_{z}$ are the rotation angles; c() and s() are abbreviations of the cosine and sine functions. The problem can be efficiently solved by the Levenberg-Marquardt algorithm [25].

### 2.4. Data Fusion Based on Gaussian Process Model

After data registration, the overlapped area of the two registered datasets were processed to form one fused dataset. Challenges exist for overlapped data fusion when the data sets are from different sources (e.g., different sensors), due to the variety of data density and uncertainty involved. In the current study, the Gaussian process (GP) model [26] was used to perform the data fusion by taking the uncertainty of the datasets into account. GP is a Bayesian regression model which can be completely specified by a mean function μ(X) and covariance function K(X,X), as given by Equation (14).

(14) f(X)~N(μ(X),K(X,X))

In the actual measurement, the zero-offset mean function was used, since no prior knowledge on the surface geometry is available. A prediction of $f^{*}$ at arbitrary location $x^{*}$ can then be given from the joint distribution of $f^{*}$ with Z as given by Equation (15) as follows:

(15) $$\left[\begin{array}{c} Z\\ f^{*} \end{array}\right]\sim N\left(\begin{array}{cc} 0, & \left[\begin{array}{cc} K\left(X,X\right)+\sigma_{\varepsilon }^{2}I & K\left(X,x^{*}\right)\\ K\left(X,x^{*}\right) & K\left(x^{*},x^{*}\right) \end{array}\right] \end{array}\right)$$

where I is the identity matrix and $\sigma_{\varepsilon }$ is a hyperparameter representing the noise variance associated in Z. A prediction m and its uncertainty cov at an arbitrary location x on the model can then be obtained from the marginal distribution of f(x) as follows [27]:

(16) $$m=K\left(x,X\right){\left(K\left(X,X\right)+\sigma_{\varepsilon }^{2}I\right)}^{-1}Z$$

(17) $$cov=K\left(x,x\right)-K\left(x,X\right){\left(K\left(X,X\right)+\sigma_{\varepsilon }^{2}I\right)}^{-1}K\left(X,x\right)$$

In the present study, the squared exponential function was used as the covariance function that provided correlation among any set of outputs. More details regarding the GP modelling, are contained in the work by Rasmussen et al. [26].

It was assumed that all the datasets at the overlapping areas possessed the same shape and hence the same GP covariance except the noise parameters, since different datasets would have different levels of uncertainties. Then, for the two datasets $Z_{1}\;\mathrm{and}\;Z_{2}$, the fused GP model was simply established by treating the fusion process as a standard GP regression problem as given by Equation (18):

(18) $$\left[\begin{array}{c} \bar{Z}\\ f_{f}^{*} \end{array}\right]\sim N\left(\begin{array}{cc} 0, & \left[\begin{array}{cc} K\left(\bar{X},\bar{X}\right)+diag(\sigma_{\varepsilon 1}^{2}I,\sigma_{\varepsilon 2}^{2}I) & K\left(\bar{X},x_{f}^{*}\right)\\ K\left(\bar{X},x_{f}^{*}\right) & K\left(x_{f}^{*},x_{f}^{*}\right) \end{array}\right] \end{array}\right)$$

where $f_{f}^{*}$ and $x_{f}^{*}$ are the prediction and its location, $\bar{Z}=\left[Z_{1},Z_{2}\right]$ are the two measured datasets, $\bar{X}=\left[X_{1},X_{2}\right]$ are the location of the measured datasets, and $\sigma_{\varepsilon 1}^{2}$ and $\sigma_{\varepsilon 2}^{2}$ are the hyperparameters representing the noise variance associated in $Z_{1}\;\mathrm{and}\;Z_{2}$. Recalling that Equation (18) is an extension of Equation (15) that the measured data contains two different levels of noise, hence the mean $m_{f}^{*}$ and the variance $cov\left(f_{f}^{*}\right)$ of the $f_{f}^{*}$ on the fused GP model can also be obtained in similar way by the marginal distribution of $f_{f}^{*}$ as given by Equation (19).

(19) $$f_{f}^{*}|x_{f}^{*},\bar{X},\bar{Z}\sim N\left(m_{f}^{*},\mathrm{cov}\left(f_{f}^{*}\right)\right)$$

## 3. Experimental Verification

### 3.1. Simulation Study

Simulation studies were undertaken to verify the proposed method for data registration and fusion. A normal surface is defined as

(20) $$\left\{ \begin{array}{l} f(x,y)=\sin(x)+\cos(y)\\ x\in [-5,5],y\in [-3,3] \end{array}\right.$$

**Figure 6:**
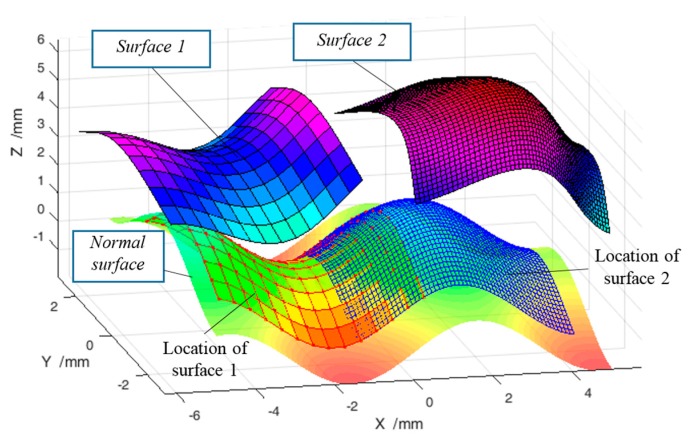
Normal surface and two extracted surfaces with different positions and postures.

As shown in Figure 6, two sub-surfaces were sampled from the designed surface with different spacings (0.4 mm and 0.1 mm)at different locations, and were denoted as Surface 1 and Surface 2. Surface 1 was extracted at x∈[−4.5,1], y∈[−2,2] and was moved to a position based on the transformation of $T\left(\frac{\pi }{20},-\frac{\pi }{15},\frac{\pi }{30},-1.5,0.5,4\right)$, based on Equation (13). Surface 2 was extracted at x∈[−1,4.5], y∈[−2,2] and was moved to a position based on the transformation of $T\left(\frac{\pi }{25},\frac{\pi }{10},\frac{\pi }{30},-1.5,-0.5,5\right)$. Surface 1 and Surface 2 had Gaussian noise added with standard deviations of 0.2 μm and 0.5 μm respectively, to represent measurement errors. It was noted that Surface 1 and Surface 2 had different resolutions with different associated uncertainties, and were embedded in different coordinate frames, which is very common in multi-sensor surface metrology in order to balance measurement efficiency and accuracy. The proposed method was then used to perform registration and fusion of the two surfaces.

**Figure 7:**
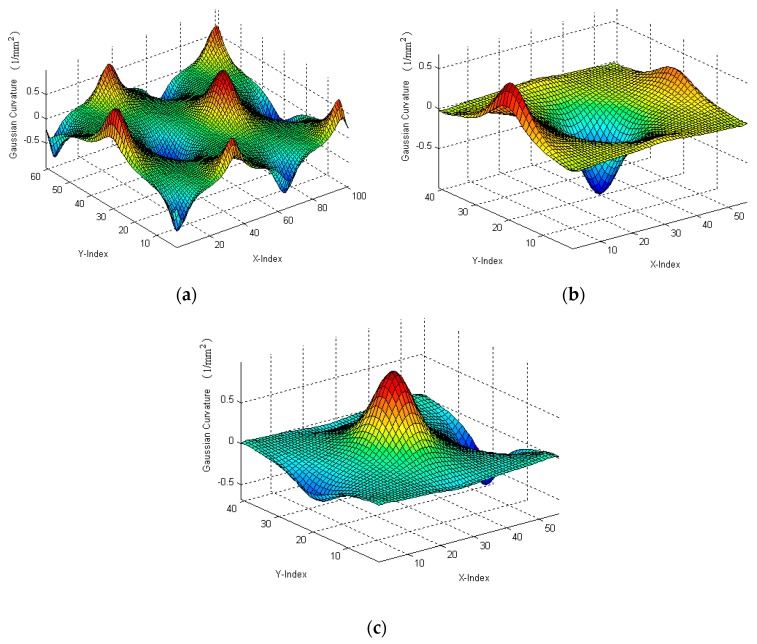
Gaussian curvatures of (**a**) normal surface; (**b**) extracted Surface 1; and (**c**) extracted Surface 2.

**Figure 8:**
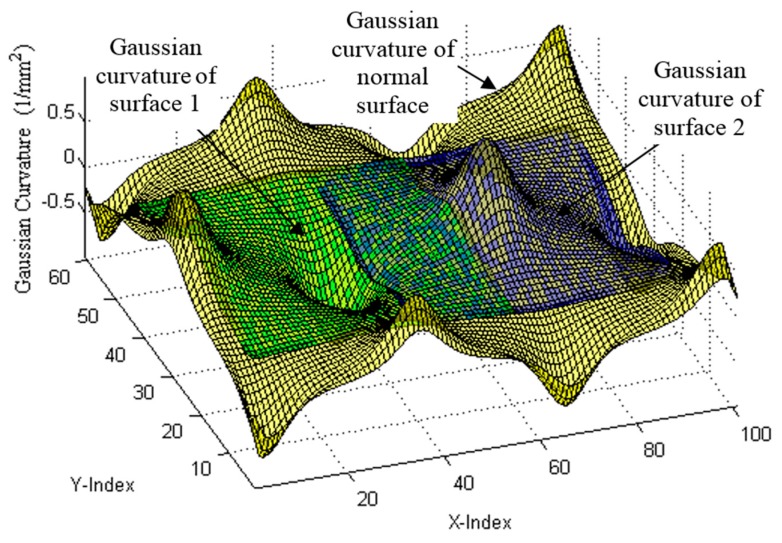
Registration of Gaussian curvatures of two sub-surfaces to normal surface.

**Figure 9:**
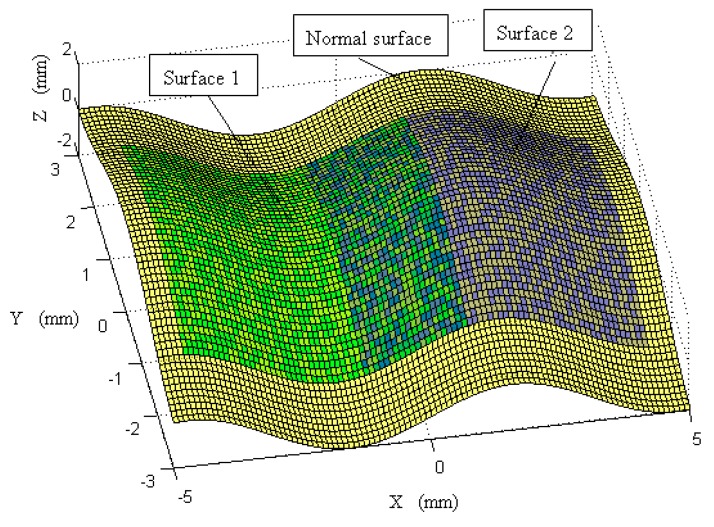
Surface registration results of the two sub-surfaces to normal surface.

Gaussian curvatures were employed as ISFs, and were calculated as shown in Figure 7. No matter where and what positions and postures the sub-surfaces are, their Gaussian curvatures remained the same. This validated that the Gaussian curvature was the ISF of the surface and is free from the coordinate frame. For the next step, surface registration was undertaken based on such ISFs. Figure 8 shows the results of registration of Gaussian curvatures of two sub-surfaces to the normal surface; while Figure 9 is the corresponding surface registration results of the two surfaces to the normal surface data. The registration process was repeated 50 times to evaluate the reliability of the proposed method. Table 1 summarizes the error of the evaluated six spatial parameters for Surface 1, by the proposed method, as well as by the classical ICP method [14]. It was interesting to note from the results that the performance of the proposed method well matched with that of the ICP method, and possessed slightly lower variance. This was due to the fact that the surface reconstruction process not only rejected the noise of the registered datasets, but also created a larger number of correspondences by ISF registration.

**Figure 10:**
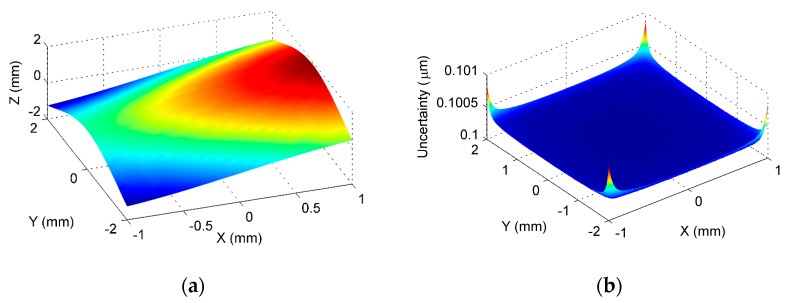
Fusion model of the overlaping area based on the Gaussian process (GP) model. (**a**) GP model at overlaping area; (**b**) Estimated uncertainty of the established GP model.

**Figure 11:**
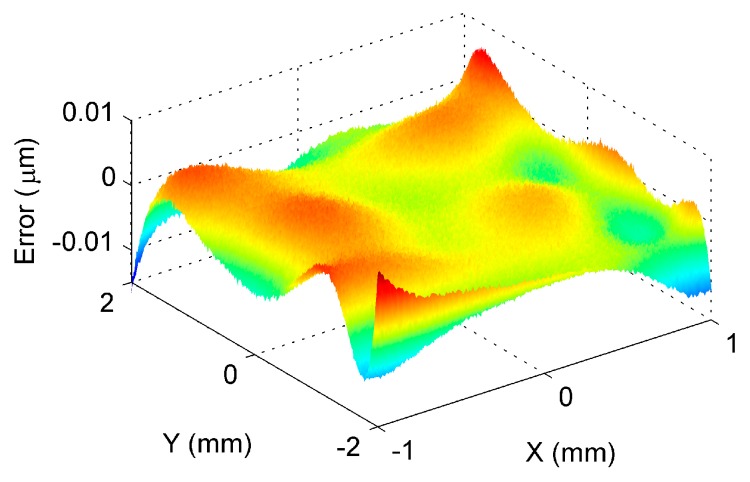
Deviation of the stitched surfaces from the designed surface.

After transforming the two surfaces into a common coordinate system, data fusion was then performed at the overlapping area based on the method presented in Section 2.4. It was emphasized that, since the proposed method performed the registration based on the ISFs, the overlapping of the two surfaces could also be easily identified in the ISF registration process. Figure 10 shows the fused GP model and its estimated uncertainty at the overlapping area. It was seen from the estimated uncertainty that the contained noise in the data was successfully estimated in the GP modelling and fusion process. Figure 11 shows the deviation of the established GP model from the designed surface, and Table 2 summarizes the evaluated peak-to-valley (PV), height error, and the root-mean-square (RMS) height error. Since no form error was added to the sampled surface, i.e., Surface 1 and Surface 2, the theoretical form error at the overlapping area should be zero. Hence, the evaluated form error should be the error resulting from the measurement noise. It was seen from the results that, based on the proposed method, the error was been reduced to several nanometers via the GP modeling and fusion process, when the magnitude of the measurement noise was at sub-micrometer level. It was clearly seen from the Table 2 that the proposed method had much better accuracy than the existing method. This means that the proposed method successfully registered the two freeform surfaces under the existence of the measurement noise, and the accuracy of the overlapping area was further improved, based on the proposed GP based data fusion method. The proposed method was also compared with an existing method which was currently widely used in practice. The existing method uses ICP to register the two surfaces, and the data at the overlapping area is fused based on the weighted mean (WM) method [16]. It is interesting to note from the comparison, that the proposed method had a much better performance than the ICP-WM method, in registering and fusing the freeform surfaces under the existence of the measurement noise.

### 3.2. Application in Actual Measurement

**Figure 12:**
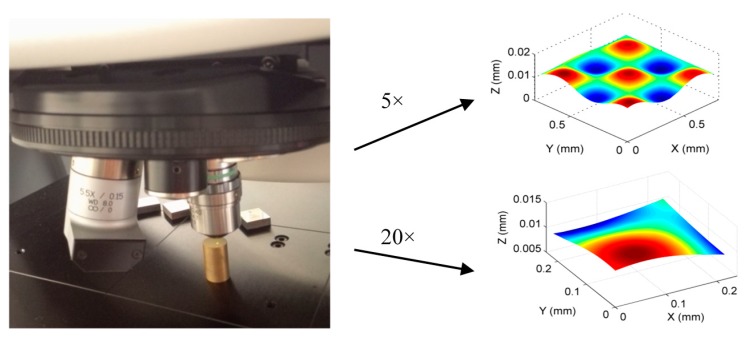
Measurement of a machined micro-structured surface.

An experimental study was conducted to evaluate the performance of the proposed method. A sinusoidal micro-structured surface defined by Equation (21) was machined by a four-axis ultra-precision machining system (Moore Nanotech 350). To fully capture the geometric information, including the surface form and texture, the workpiece was measured by a 3D optical profiler (Zygo NexView) with two different objectives i.e., 5.5× and 20× magnifications (zoom was 2.0×). The field of view of the two objectives were 0.76 × 0.76 mm^2^ and 0.21 × 0.21 mm^2^, and the maximum slopes of the two objectives were 7.27°and 21.80°, respectively. The measured data are shown in Figure 12.

(21) z=0.015*(sin(15x)+cos(15y))

**Figure 13:**
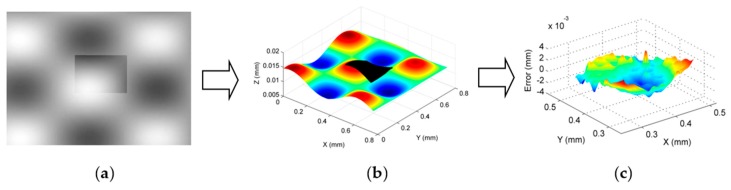
Datasets registration and fusion based on the proposed method. (**a**) ISF registration; (**b**) Datasets after registration; (**c**) Evaluated form error.

The two datasets had different resolutions and were embedded in two different coordinate frames, since they were measured by two different objectives in two steps. As a result, the proposed method was used to perform data stitching and fusion, so as to obtain a unique representation of the machined surface. It started by registering the dataset measured by the 5.5× and 20× objectives to the nominal surface, using the proposed ISF based data stitching method. Filtering was carried out to remove outliers and noise before the calculation of the intrinsic surface feature. Figure 13a shows the registration of the ISFs of the two datasets, and Figure 13b shows the registered two datasets. After data registration, the overlapped area between the two datasets was processed based on the proposed fusion method. To examine the quality of the result, comparison was made between the evaluated form error of the overlapped area on the datasets obtained by 5.5× and 20× objectives, and the fused dataset. Figure 13c shows the form error evaluated by the fused dataset. The RMS errors evaluated by the three datasets were 0.011 µm, 0.097 µm, and 0.087 µm respectively. It is interesting to note from the results that the RMS errors obtained by fused dataset matched well with those obtained by the 5.5× and 20× objectives, which implied that the registration and fusion had been carried out accurately.

**Figure 14:**
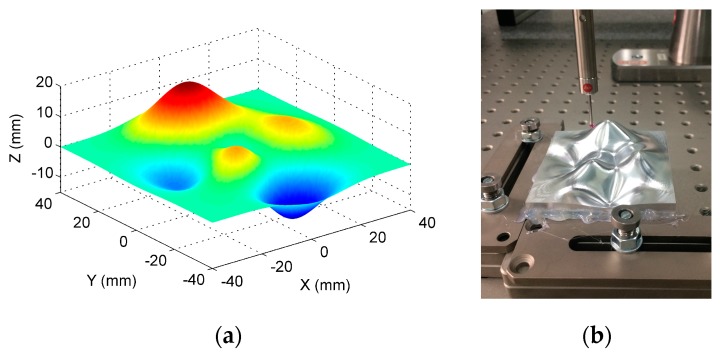
Measurement of a general freeform surface. (**a**) Designed freeform surface; (**b**) Machined surface on a coordinate measuring machine (CMM).

To further evaluate the capability of the proposed method, an actual measurement was conducted on another general freeform surface. Figure 14 shows the machined freeform surface which was designed by the peak function and contained several peaks and valleys, and was a representative type of freeform surface.

**Figure 15:**
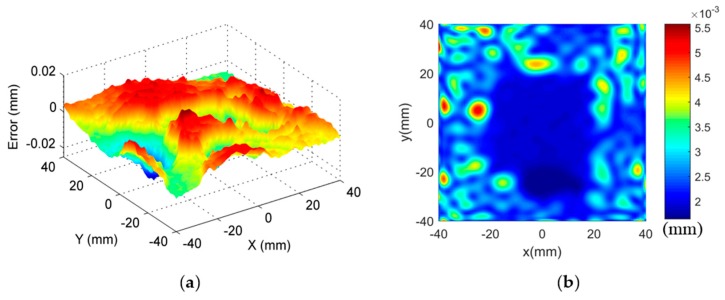
Form error evaluation results. (**a**) Evaluated form error; (**b**) Uncertainty of the fused GP model.

The combined use of large scale fast inspection sensors, e.g., laser scanner and photogrammetry, with micro scale pointwise measuring sensors, e.g., coordinate measuring machines, are currently the most common scenarios for multi-sensor surface metrology. Therefore, in the present study, a high precision coordinate measuring machine (CMM) and a laser scanner were combined to measure the freeform surface for efficient and reliable measurement. The CMM possessed a length measurement uncertainty with U = (0.6 + L/500, L in mm) μm, and a probing error with u = 0.9 μm (1σ, normal). The uncertainty of the laser scanner was identified to be u = 3.4 μm (1σ, normal) by a reference ball. The measurement was carried out in two steps. Firstly, CMM was used to measure the surface with spacing 1 mm in both X and Y directions. A total of 6456 points were uniformly sampled with 1 mm spacing over the entire surface. Figure 15 shows the form error evaluation results. The PV and RMS values of the measured surface were found to be 34.9 μm and 5.5 μm. The values served as a benchmark to verify the effectiveness of the proposed method.

Secondly, multi-sensor measurement strategy was carried out. CMM was used to measure the surface with 4 mm spacing in both X and Y directions. Laser scanning was used to perform dense measurements of the surface. The proposed method was then used to process the measured datasets. ISF-based data registration method was used to precisely register the measured datasets by the two sensors into a common coordinate system. The datasets at the identified overlapping area were then fused based on the GP model. To verify the effectiveness of the proposed method, only the overlapping part was used for the form error evaluation of the machined surface. A summary of the experimental results is shown in Table 3. Both the evaluation parameters and the time consumption for the measurement are given to analyze both the efficiency and accuracy of the proposed method.

As a fast measurement sensor, the laser scanner has the highest measurement efficiency, while its accuracy is relatively low compared with CMM. For CMM measurements with loose sampling, the measurement time became acceptable, while the inadequate sampling of the surface would underestimate the form error of the machined surface, especially the PV, which was determined by extreme points. Hybrid measurement on the other hand, had the best overall performance. It was shown from results that, by registering and fusing the datasets from the laser scanner and the CMM, accuracy was dramatically improved, while the measurement efficiency was maintained as well. The experiment verified the capability of the multi-sensor measurement in freeform surface measurement, and further confirmed the effectiveness of the proposed method in addressing the key issues in the measurement process.

## 4. Conclusions

Freeform data stitching and fusion technology provides a practical solution for multi-sensor measurement of freeform surfaces, and also for enhancing the measuring ability of some high precision measurement instruments. This paper presented methods and algorithms for data stitching and fusion for measuring ultra-precision freeform surfaces based on the registration of ISFs. Some important algorithms involved in the registration algorithms were explained, including data format unification, calculation of ISFs such as Gaussian curvatures and mean curvatures, registration of the ISF map, Gaussian process-based data fusion for the overlapping area, etc. Experimental studies were conducted and the results were discussed. The proposed method and algorithms of data stitching and fusion intend to eliminate or alleviate the effect of noise and outliers, and provide a robust registration and fusion method, which is helpful for multi-scale and multi-sensor measurement of ultra-precision freeform surfaces.

## Figures and Tables

**Table 1:** A summary of the error of the evaluated six spatial parameters.

	*r_x_* (μrad)	*r_y_* (μrad)	*r_z_* (μrad)	*t_x_* (nm)	*t_y_* (nm)	*t_z_* (nm)
ISFM	1.7/0.5 *	4.2/1.9	3.9/1.2	9.9/3.2	7.8/1.9	3.5/0.8
ICPM	2.1/0.6	5.5/2.2	3.7/1.6	12.8/6.3	9.8/2.5	3.4/0.8

* *a*/*b*: *a* refers to mean error, *b* refers to the variance. ISFM: Intrinsic surface features based method. ICPM: Iterative closest point method.

**Table 2:** A comparison of the ISF+GP method with existing method.

	Fusion by ISF + GP	Fusion by ICP + WM
RMS (nm)	2	7
PV (nm)	24	78

RMS: Root mean square. PV: Peak-to-valley. WM: Weighted mean. ISF: Intrinsic surface features. ICP: Iterative closest point.

**Table 3:** A summary of the experimental results.

	PV	RMS	Time (h)
Benchmarking	36.8	5.7	>3
Laser scanner	49.6	8.1	<0.16
CMM	33.2	4.8	~0.5
Hybrid	35.4	5.5	~0.5
